# Impact of Biologic and Targeted Synthetic Therapies on Retinal and Choroidal Parameters Assessed by Optical Coherence Tomography Angiography

**DOI:** 10.3390/biomedicines14081753

**Published:** 2026-08-04

**Authors:** Ilona Katarzyna Jędrzejewska, Marta Łosoś, Anna Felis-Giemza, Joanna Gołębiewska

**Affiliations:** 1 Ophthalmology Center “Świat Oka”, 02-511 Warsaw, Poland; ilona.88@wp.pl; 2 National Institute of Geriatrics, Rheumatology and Rehabilitation, 02-637 Warsaw, Poland; annafelis@wp.pl; 3 Department of Ophthalmology, Military Institute of Aviation Medicine, 01-755 Warsaw, Poland; joanna.golebiewska@wp.pl

**Keywords:** axial spondyloarthritis, optical coherence tomography angiography, retinal microcirculation, foveal avascular zone, TNF inhibitors, IL-17 inhibitors, JAK inhibitors, smoking

## Abstract

**Background**: To compare retinal microvascular parameters assessed by optical coherence tomography angiography (OCTA) among patients with axial spondyloarthritis (axSpA) receiving different biologic and targeted synthetic therapies and to evaluate the association between smoking status and OCTA-derived retinal microvascular parameters. **Methods**: This cross-sectional study included 159 eyes of 82 patients with radiographic or non-radiographic axSpA receiving tumor necrosis factor inhibitors (TNFi), interleukin-17 inhibitors (IL-17i), or Janus kinase inhibitors (JAKi) for at least one year. At the time of examination, all patients had BASDAI scores below the established threshold for active disease (BASDAI < 4). Comprehensive ophthalmic evaluation and swept-source OCTA imaging were performed. Superficial capillary plexus vessel density and foveal avascular zone (FAZ) areas in both the superficial (SCP) and deep capillary plexus (DCP) were assessed. Statistical analyses were adjusted for age, sex, smoking status, history of uveitis, treatment duration, and ocular variables. **Results**: Significant differences were observed among treatment groups in the deep foveal avascular zone (DCP-FAZ) area (*p* = 0.0060). Post hoc analyses demonstrated larger DCP-FAZ values in patients receiving TNFi than in those receiving IL-17i (*p* = 0.0010), and larger values in the IL-17i group than in the JAKi group (*p* = 0.0151). No significant differences were observed in superficial FAZ area or superficial capillary plexus vessel density between treatment groups. Patients receiving JAKi were older and had a shorter treatment duration than those in the other groups. Smoking status was associated with a significantly smaller deep FAZ area (*p* = 0.0019), whereas no significant association was observed for superficial FAZ measurements. A weak positive correlation was found between intraocular pressure and deep FAZ area (r = 0.18, *p* = 0.0237), and treatment duration was weakly positively correlated with deep FAZ area (rho = 0.16, *p* = 0.0454). **Conclusions**: Retinal microvascular parameters differed among patients with axSpA receiving different classes of biologic and targeted synthetic therapies. Differences were observed primarily in the deep FAZ area, while smoking status was also associated with deep retinal microvascular parameters. Because of the cross-sectional design, these findings should be interpreted as associations rather than evidence of treatment effects. OCTA may provide a useful non-invasive method for assessing retinal microvascular characteristics in patients with axSpA. Further longitudinal studies are warranted to clarify the relationship between systemic therapies, smoking status, and retinal microvascular changes.

## 1. Introduction

Axial spondyloarthritis (axSpA) is a chronic inflammatory rheumatic disorder primarily affecting the axial skeleton, particularly the spine and sacroiliac joints [1]. In addition to musculoskeletal involvement, the disease is frequently associated with extra-articular manifestations, including uveitis, psoriasis, and inflammatory bowel disease (IBD) [2].

Based on imaging findings, axSpA is classified into radiographic axSpA (r-axSpA), formerly known as ankylosing spondylitis, and non-radiographic axSpA (nr-axSpA). Although these subtypes differ in the presence of structural sacroiliac joint changes visible on conventional radiography, they share similar clinical manifestations, disease burden, and treatment strategies [2,3,4].

Among extra-articular manifestations, acute anterior uveitis is one of the most common and affects approximately 6–30% of patients, particularly those who are HLA-B27 positive [5].

The diagnosis of axSpA is currently based on the Assessment of SpondyloArthritis international Society (ASAS) classification criteria [3].

Disease activity is commonly assessed using the Ankylosing Spondylitis Disease Activity Score (ASDAS) and the Bath Ankylosing Spondylitis Disease Activity Index (BASDAI). The primary treatment goal in axSpA is remission or, alternatively, low disease activity [4].

In the pathogenesis of axSpA, the pro-inflammatory cytokines interleukin-17 (IL-17) and tumor necrosis factor alpha (TNF-α) play a role [6,7]. In patients with axSpA, endothelial dysfunction occurs even in the early stages of the disease [8]. In the vasculature, IL-17 promotes the production of proinflammatory cytokines, chemokines and adhesion molecules, enhances the effects of TNF and leukocyte trafficking and recruitment. As a consequence, an elevated level of this cytokine may contribute to vascular inflammation and is implicated in endothelial dysfunction [9].

Tumor necrosis factor alpha (TNF-α) is a proinflammatory cytokine that may impair endothelial function and promote oxidative stress [7].

Janus kinases (JAKs) are a family of intracellular tyrosine kinases that act as signal transducers, mediating the transmission of extracellular cytokine signals and activating downstream proteins involved in cellular signaling pathways (JAK-STAT). The JAK–STAT proteins amplify the effects of pro-inflammatory cytokines involved in the pathogenesis of various diseases, including axSpA [10]. By inhibiting JAK–STAT signaling, JAK inhibitors may protect the vascular endothelium through the reduction in IL-6-mediated inflammatory responses induced by TNF and IL-17A. 

The introduction of biologic therapies, such as TNF-α inhibitors (TNFi) and IL-17 inhibitors (IL-17i), as well as targeted synthetic disease-modifying drugs, such as JAK inhibitors (JAKi), has significantly improved the treatment of systemic disease. TNFi alleviates both systemic and local inflammation, reduces vascular permeability, inhibits endothelial activation, and helps prevent long-term tissue damage. IL-17i alleviates inflammation by regulating neutrophil recruitment, while JAKi disrupts intracellular cytokine signaling, resulting in broad immunomodulatory effects [11,12].

Recent recommendations continue to support TNF inhibitors and IL-17 inhibitors as the preferred biologic therapies for patients with active axSpA who do not respond adequately to NSAIDs, whereas JAK inhibitors may be considered in subsequent lines of therapy [2,13,14].

Studies have shown that biologic therapies (TNFi and IL-17i) as well as targeted synthetic (JAKi) demonstrate significant efficacy in both radiographic and non-radiographic spondyloarthritis. These treatments reduce inflammation and significantly improve patients’ quality of life. The meta-analysis by Li et al. suggests that, in patients with non-radiographic axial spondyloarthritis, TNF inhibitors (TNFi) may be slightly more effective than other therapeutic classes. Nevertheless, all treatment groups were associated with significantly higher ASAS40 response rates compared with the placebo [15,16].

Although these therapies are primarily evaluated for clinical efficacy and systemic safety, their potential impact on retinal microvascular parameters remains incompletely understood.

Cigarette smoking is a well-established cardiovascular risk factor that has also been associated with retinal microvascular alterations. Previous OCTA studies have demonstrated associations between smoking and enlargement of the FAZ, reduced retinal vessel density, and impaired retinal perfusion. Therefore, smoking status may represent an important factor influencing OCTA-derived retinal microvascular parameters.

Optical coherence tomography angiography (OCTA) is a non-invasive imaging method that provides highly detailed, three-dimensional images of the entire microcirculation of the retina and choroid, enabling an assessment of retinal blood flow. This method allows for the identification of retinal vessels by detecting and measuring the movement of blood cells within them without the need for dye injection [17,18].

Given the close relationship between systemic inflammation and retinal microcirculation, OCTA-derived parameters may provide objective biomarkers of treatment-related changes in retinal structure and perfusion. Changes in the foveal avascular zone (FAZ) and density of the superficial and deep capillary plexus may indicate disturbances in the vascular integrity of the retina.

The aim of this study was to compare retinal microvascular parameters in patients with axSpA receiving TNFi, IL-17i or JAKi and to evaluate the association between smoking status and OCTA-derived retinal microvascular parameters.

## 2. Material and Methods

This cross-sectional study was conducted between May 2025 and March 2026 at the Świat Oka Eye Center in Warsaw, Poland. The study included 159 eyes of 82 patients diagnosed with axSpA receiving biological and targeted synthetic disease-modifying drugs: TNFi, IL-17i or JAKi. The participants were referred from the Biological Therapy Centre of the National Institute of Geriatrics, Rheumatology and Rehabilitation. The study protocol was approved by the Bioethics Committee at the National Institute of Geriatrics, Rheumatology and Rehabilitation named after Prof. Eleonora Reicher, and was carried out in accordance with the tenets of the Declaration of Helsinki. 

The inclusion criteria were a diagnosis of axial spondyloarthritis (radiographic or non-radiographic form), biological treatment for at least 1 year (including TNFi, IL-17i, JAKi),and age between 20 and 50 years. Exclusion criteria were myopia (≥3.0 Dpsh), hyperopia (≤3.0 Dpsh), active uveitis, glaucoma, ocular hypertension, history of intraocular or intracranial surgery, history of optic neuritis, history of optic disc oedema, diabetic retinopathy, uncontrolled arterial hypertension, hereditary retinal degeneration, and lack of written consent to participate in the study. Eyes with poor-quality OCTA images, segmentation errors, or significant motion artifacts were also excluded from the analysis. 

All patients met the Assessment of SpondyloArthritis international Society (ASAS) criteria. AxSpA patients were divided into three groups (TNFi, IL-17i, JAKi) in further analyses. At the time of examination, all patients had BASDAI scores below the established threshold for active disease (BASDAI < 4).

Patients received biological or targeted synthetic disease-modifying therapies according to current clinical practice and approved dosing regimens. TNFi included adalimumab (40 mg administered subcutaneously every 2 weeks), certolizumab pegol (200 mg administered subcutaneously every 2 weeks), etanercept (50 mg administered subcutaneously once weekly), and golimumab (50 mg administered subcutaneously once monthly). IL-17i included secukinumab (150 or 300 mg administered subcutaneously once monthly) and ixekizumab (80 mg administered subcutaneously every 4 weeks). JAKi included upadacitinib (15 mg orally once daily) and tofacitinib (5 mg orally twice daily).

Study participants underwent a complete ophthalmic examination, including slit lamp biomicroscopy with dilated fundus examination using a 90-diopter (D) lens, best-corrected visual acuity (BCVA) measured using a Snellen chart, refractive error, and intraocular pressure measurement (non-contact tonometry using autokeratorefractotonometr TOPCON TRK-2P, Topcon Corporation, Tokyo, Japan).

**Figure 1:**
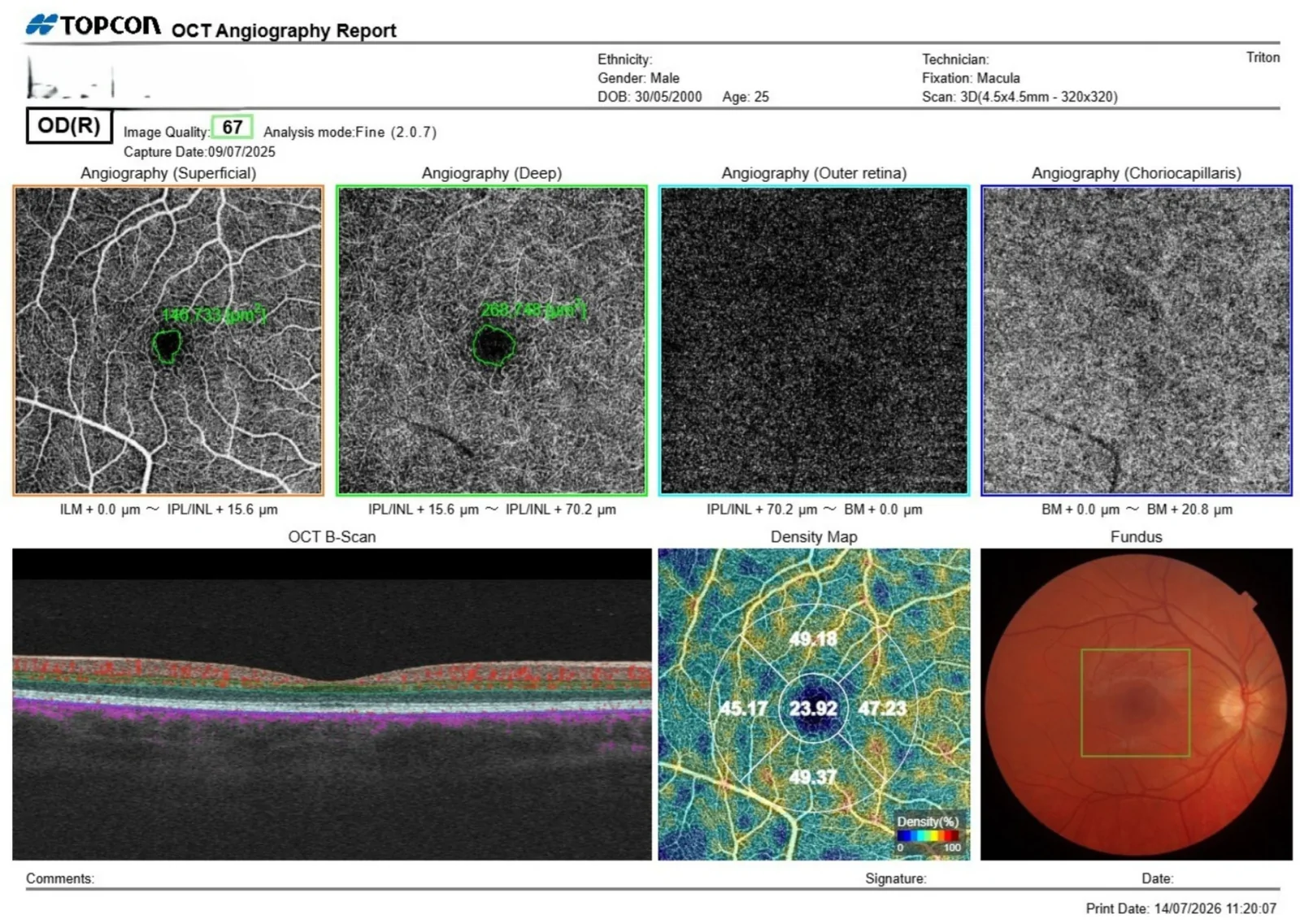
Representative swept-source OCTA image of the right eye (OD) from a patient with axSpA receiving an IL-17i. Representative images of the SCP, DCP, outer retina, choriocapillaris, OCT B-scan, vessel density map, and color fundus photograph are shown. The manually delineated FAZ is outlined in green in both the SCP and DCP images. The numerical values displayed on the OCTA images represent automatically generated OCTA measurement parameters provided by the device software.

**Figure 2:**
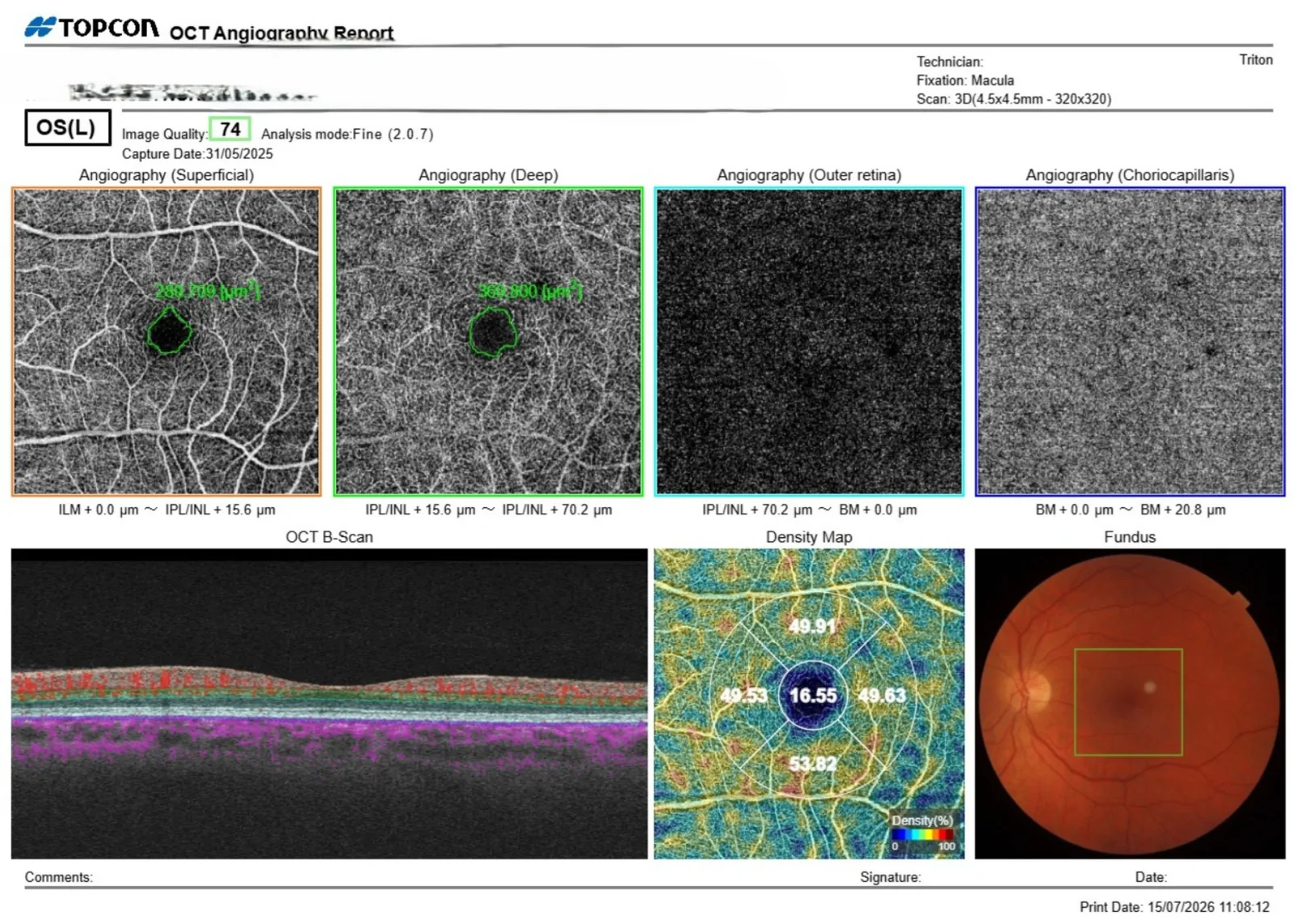
Representative swept-source OCTA image of the left eye (OS) from a patient with axSpA receiving a TNFi. Representative images of the SCP, DCP, outer retina, choriocapillaris, OCT B-scan, vessel density map, and color fundus photograph are shown. The manually delineated FAZ is outlined in green in both the SCP and DCP images. The numerical values displayed on the OCTA images represent automatically generated OCTA measurement parameters provided by the device software.

**Figure 3:**
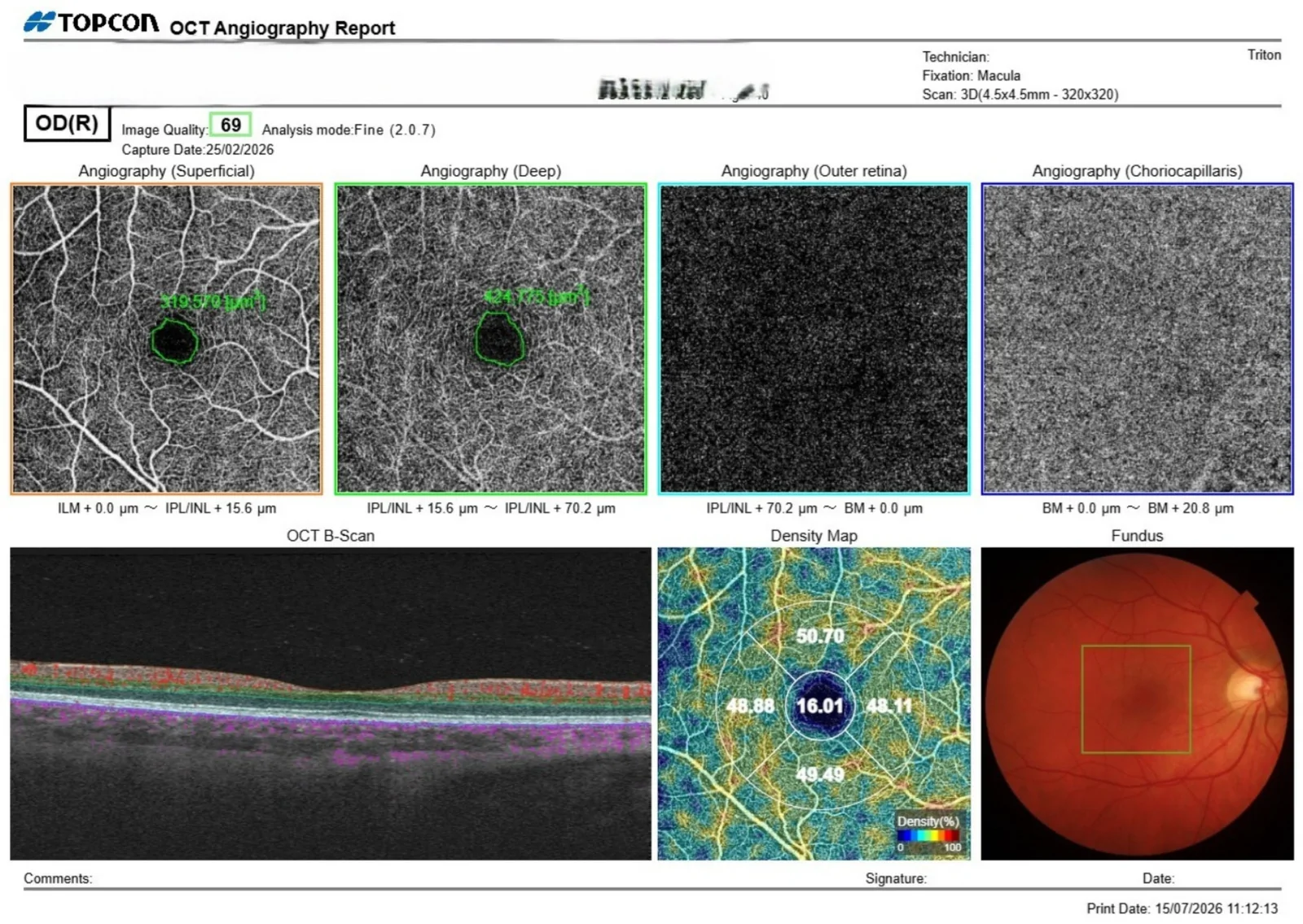
Representative swept-source OCTA image of the right eye (OD) from a patient with axSpA receiving a JAKi. Representative images of the SCP, DCP, outer retina, choriocapillaris, OCT B-scan, vessel density map, and color fundus photograph are shown. The manually delineated FAZ is outlined in green in both the SCP and DCP images. The numerical values displayed on the OCTA images represent automatically generated OCTA measurement parameters provided by the device software.

Multimodal imaging was performed using a Swept-Source OCT (SS-OCT) device (Topcon DRI OCT Triton, Topcon Corporation, Tokyo, Japan). The OCTA was performed using 4.5 × 4.5 mm images centered on the macula. On OCTA images, capillary plexus density (CPD) of the vessels in the parafoveal and perifoveal regions was evaluated in the superficial capillary plexus (SCP). The scan automatically inserted fovea-centered circles at the macula, detecting SCP. The SCP network was segmented from 3 μm beneath the internal limiting membrane to 15 μm below the inner plexiform layer (IPL). The foveal vessel density was defined as the area of the small circle, with a diameter of 1 mm. The parafoveal vessel density was defined as the area of the outer circle. It was automatically divided into four quadrants: superior (S), inferior (I), nasal (N), and temporal (T). The vessel density was automatically calculated as a percentage, according to the area occupied by blood vessels, by the software. The average vessel density of the SCP in the four quadrants was calculated. The software used does not measure vascular density in the DCP. The FAZ area was manually measured by the same examiner in both the SCP and DCP using the built-in software of the Topcon DRI OCT Triton (IMAGEnet 6 version 1.04E, Topcon Corporation, Tokyo, Japan). FAZ values are reported in square micrometres (µm^2^), according to the units displayed by the device software. Two scans for each eye were captured, and the highest-quality scan was selected for analysis. Representative OCTA images of the central macula from patients receiving TNFi, IL-17i, and JAKi are presented in Figure 1, Figure 2 and Figure 3 to illustrate the retinal microvascular images analyzed in this study.

All participants were examined at a similar time of day, between 11:00 AM and 1:00 PM. Information on smoking status and history of uveitis was collected for all participants.

All patients met the classification criteria for the Assessment of SpondyloArthritis International Society (ASAS) criteria [12]. AxSpA patients were divided into two subgroups (r- and nr- axSpA) in further analyses. Forty-three of 70 patients with axSpA were treated with anti TNF-a therapy. Disease activity was assessed using the Bath Ankylosing Spondylitis Disease Activity Index (BASDAI) and the Ankylosing Spondylitis Disease Activity Score (ASDAS)-CRP. None of our patients had active anterior uveitis during the measurements.

Informed consent was obtained from all subjects involved in the study.

### Statistical Procedures

Categorical variables were shown as integer numbers and percentages (frequencies). Numerical traits were described through their mean, median, standard deviation, standard error, 95% confidence interval, and lower-to-upper quartile values. The normality of distribution was assessed by using the Shapiro–Wilk W test. Levene’s test was used to test the homogeneity of variances. A multifactor analysis of variance (ANOVA) without replications was carried out in order to test the differences in normally distributed variables. Generalized linear models (GZM) were fitted when dealing with non-normally distributed variables or heterogeneous variances. All the multifactor procedures were controlled for the study participants’ age, gender, smoking status, history of uveitis, biological treatment duration, relevant ophthalmic covariates, including axial length and intraocular pressure. In some models, the cofactors mentioned naturally became dependent variables to complete the planned statistical analyses. The Pearson product-moment correlation coefficients were obtained when assessing relationships between numerical traits. Spearman’s rank correlation was fitted for the treatment duration due to its strong non-normal distribution. The Tukey–Kramer *post hoc* test for pairwise comparisons was performed following significant omnibus analyses. Considering that multiple OCT outcomes were evaluated, the analyses should be interpreted as exploratory in nature.

Because both eyes from a number of participants were included in the analyses, an error-correction approach accounting for intra-subject correlation between fellow eyes was applied where appropriate. Therefore, all the statistical tests involving ophthalmological parameters, except the study participants’ characteristics such as their age, gender, disease duration and data on their smoking habit, were carried out by using the error correction modality of clustering outcomes by unique patient number. The independent correlation structure was set due to the lack of repeated measurements. A level of *p* < 0,05 was considered statistically significant. All the procedures were carried out in Stata, release 14.2 (StataCorp LLC, College Station, TX, USA).

## 3. Results

The study included 159 eyes of 82 patients with axSpA receiving biologic or targeted synthetic disease-modifying therapy with a mean treatment duration of 44.33 months. Among the analyzed patients, 32 individuals (39.02%) were treated with TNFi. The most frequently used agent was adalimumab, administered to 16 patients (19.51%), followed by golimumab in 9 patients (10.98%), etanercept in 4 patients (4.88%), and certolizumab pegol in 3 patients (3.66%). IL-17i was used in 30 patients (36.59%). Within this group, secukinumab was administered to 26 patients (31.70%), whereas ixekizumab was used in 4 patients (4.88%). JAKi were administered to 20 patients (24.39%). upadacitinib was used in 19 patients (23.17%), while tofacitinib was administered to 1 patient (1.22%) (Table 1).

The demographic and basic clinical characteristics of the groups are presented in Table 1.

The structure of the three therapeutic groups by gender was statistically significantly different (*p* = 0.0269). In the study group with IL-17i, men clearly outnumbered women. Male patients also prevailed in the group with JAKi. The discussed disproportions were less pronounced in the group with TNFi (Table 2).

Study participants administering TNFi reported uveitis statistically significantly more frequently than other subjects (*p* = 0.0027) (Table 2).

The study participants who were administering JAKi were statistically older than the remaining two groups (*p* = 0.0389). The age of participants taking TNFi and IL-17i was equalized (Table 2).

Statistically significant differences were observed in the deep foveal avascular zone between the study groups (*p* = 0.0060). Post hoc tests showed significant differences between the group with TNFi vs. IL-17i (*p* = 0.0010) and IL-17i vs. JAKi (*p* = 0.0151) (Table 2).

The treatment duration was statistically significantly shorter in the group with JAKi (*p* = 0.0007) (Table 2).

There were no statistically significant differences in superficial capillary plexus vessel density between the TNFi, IL-17i, and JAKi treatment groups. Comparable values were observed for the central region and all parafoveal quadrants (superior, inferior, nasal, and temporal), with all comparisons yielding non-significant results (*p* > 0.05) (Table 3).

Significant differences were observed between smokers and non-smokers within the study population. The superficial foveal avascular zone did not differ statistically significantly in the study participants by smoking status (*p* = 0.2303). However, the deep FAZ revealed a large difference between the smokers and the non-smokers (*p* = 0.0019) (Table 4).

The biological treatment duration was statistically significantly longer in the study participants who smoked as compared to the non-smokers (*p* = 0.0386) (Table 4).

A weak positive correlation was observed between intraocular pressure and deep FAZ area (r = 0.18, *p* = 0.0237). Treatment duration demonstrated a weak positive monotonic correlation with deep FAZ (rho = 0.16, *p* = 0.0454).

Selected, statistically significant, Pearson correlation *r* coefficients for the examined eyes:

Ocular tension and deep FAZ, *r* = 0.18 (*p* = 0.0237).

Selected, statistically significant Spearman’s rank correlation *rho* coefficients for the examined eyes:

Treatment duration and deep FAZ, *rho* = 0.16 (*p* = 0.0454).

Baseline differences between treatment groups, particularly regarding age, sex distribution, history of uveitis, and treatment duration, should also be considered when interpreting these findings. Although these variables were included as covariates in the statistical models, residual confounding cannot be completely excluded because of the observational nature of the study.

## 4. Discussion

In this study, we observed significant differences between the treatment groups in retinal microvascular parameters, including the FAZ area in the DCP. These findings suggest that retinal microvascular parameters may differ among patients receiving biologic therapies and targeted synthetic disease-modifying drugs. To the best of our knowledge, this is the first study to directly compare the effects of different classes of these therapies on selected parameters of retinal microcirculation. Importantly, because of the cross-sectional design of the present study, the observed differences should be interpreted as associations rather than evidence of causal treatment effects.

Recent studies have highlighted the importance of assessing retinal microcirculation in patients with rheumatic diseases. Previous studies have reported that patients with rheumatic diseases may exhibit subclinical retinal microvascular abnormalities, even in the absence of clinically apparent cardiovascular disease. The increased retinal venular diameter observed in these patients may reflect early vascular dysfunction associated with chronic systemic inflammation [15].

Several studies on inflammatory rheumatic diseases have reported an association between increased retinal venular diameter and higher disease activity, suggesting possible systemic microvascular involvement related to chronic inflammation. Interestingly, these changes were observed regardless of CRP levels, which may indicate that retinal microvascular alterations reflect inflammatory burden more sensitively than conventional laboratory markers [16].

Previous studies in rheumatoid arthritis, which shares several pathogenic mechanisms with axSpA, have reported an association between effective immunomodulatory therapy and improvements in retinal microvascular abnormalities accompanied by reduced systemic disease activity. In particular, treatment with a biologic DMARD was associated with a significant reduction in the wall-to-lumen ratio of retinal arterioles after 12 months, suggesting partial reversibility of inflammation-related microvascular remodeling [17].

A study published in 2020 reported that patients with ankylosing spondylitis exhibited subtle retinal microvascular abnormalities, including altered vascular tortuosity and increased retinal vascular density, which may indicate early vascular dysfunction associated with an increased cardiovascular risk. Some of these abnormalities were more pronounced in male patients, suggesting a possible association between sex and retinal microvascular changes. [18].

These findings are consistent with the concept that assessment of retinal vessels may provide valuable insight into subclinical vascular dysfunction in systemic inflammatory diseases and may serve as a sensitive biomarker of microvascular involvement. They also suggest that the observed differences in retinal microvascular parameters may be associated with systemic inflammatory status; however, the cross-sectional design of the present study does not allow conclusions regarding treatment effects or causal relationships.

Previous studies using OCT angiography have reported reduced capillary density in the macular region of patients with axSpA, consistent with the presence of subclinical retinal microvascular abnormalities. Importantly, these changes were associated with longer disease duration rather than current disease activity, suggesting a possible cumulative relationship between disease duration and retinal microcirculatory alterations [19]. However, in our study, no significant differences in superficial capillary plexus vessel density were observed between the treatment groups, whereas differences were found only in the FAZ area of the deep capillary plexus. These findings could represent differences among the treatment groups; however, because of the cross-sectional design, it cannot be determined whether they are related to treatment exposure, disease duration, or other baseline characteristics.

Several studies have reported that patients with axSpA exhibit subclinical retinal microvascular changes, including enlargement of the FAZ and reduced vessel density in both the SCP and DCP, despite ongoing treatment. Previous reports have also indicated that these microvascular abnormalities may persist in patients receiving TNFi, suggesting that chronic inflammation may continue to influence retinal microcirculation despite systemic treatment [20]. In our study, differences in the FAZ area in the DCP were observed between the treatment groups. These findings may reflect differences among the study groups; however, because of the cross-sectional design, it cannot be determined whether they are related to treatment exposure, underlying disease characteristics, or baseline differences between the groups. 

One of the ophthalmic manifestations of axSpA may be non-infectious choroiditis. Other researchers have shown that this condition, and particularly posterior choroiditis, is associated with a significant enlargement of the FAZ area of the DCP, regardless of the presence of macular edema. In contrast, anterior uveitis seems to affect the parameters of this zone mainly in cases accompanied by macular edema, whereas eyes without edema do not differ significantly from those of healthy individuals in the control group. These findings suggest that the DCP is particularly susceptible to inflammatory damage [19].

In our study, differences in the deep FAZ area were observed between the treatment groups. These findings may reflect differences in retinal microvascular involvement among the treatment groups. However, because of the cross-sectional design, no conclusions can be drawn regarding whether these differences are attributable to treatment, residual inflammation, or other disease-related factors.

The clinical significance of the observed differences in the deep FAZ remains uncertain. However, the deep capillary plexus is considered particularly vulnerable to microvascular impairment, and recent OCTA studies in biologic-treated patients with ankylosing spondylitis have demonstrated that alterations within the deep vascular complex may persist despite systemic therapy. These observations suggest that OCTA-derived parameters of the deep retinal microvasculature may provide complementary information on subclinical retinal involvement. Future longitudinal studies are required to determine whether deep FAZ alterations are associated with disease progression, visual outcomes, or response to therapy [20].

If confirmed in longitudinal studies, deep FAZ assessment may complement conventional ophthalmic evaluation by identifying patients with subclinical retinal microvascular involvement who could benefit from closer ophthalmic follow-up.

At present, these findings should not be interpreted as supporting routine OCTA screening in all patients with axSpA but rather as highlighting a promising area for future research.

Our study also identified significant differences according to smoking status. Although no significant differences in the superficial FAZ were observed between smokers and non-smokers, a significant difference was found in the deep FAZ. This observation may suggest that smoking status is associated with retinal microvascular differences in the deep capillary plexus; however, the cross-sectional design precludes conclusions regarding a causal relationship.

Previous large population-based studies have reported that cigarette smoking is independently associated with reduced vessel density in the deep capillary plexus, with evidence of a dose-dependent relationship. The present findings suggest an association between smoking and retinal microvascular alterations, particularly in the deeper vascular layers, although the underlying mechanisms remain incompletely understood [21].

In the context of our findings, these observations are consistent with an association between smoking status and retinal microvascular alterations. However, because of the cross-sectional design of the present study, no conclusions can be drawn regarding a causal relationship, and the observed associations may also be influenced by differences in study populations and concomitant systemic therapies.

Previous studies have reported that long-term smoking is associated with retinal and choroidal microvascular alterations, including increased DCP vessel density despite the absence of significant differences in SCP vessel density or overall choroidal thickness. In addition, cumulative smoking exposure has been reported to be negatively correlated with choroidal thickness, suggesting a potential association between long-term smoking and ocular vascular changes [22].

Taken together, these findings indicate that the reported associations between smoking and ocular microcirculation are complex and may differ across studies, possibly owing to differences in study populations, smoking exposure, and methodological approaches.

Some authors have reported that long-term smoking is associated with enlargement of the FAZ area and alterations in retinal vessel density, supporting an association between smoking and retinal microvascular changes [23].

Collectively, these findings are consistent with an association between long-term smoking and structural as well as vascular retinal alterations. However, the underlying biological mechanisms remain incompletely understood and cannot be inferred from the present cross-sectional study.

Importantly, the cross-sectional nature of the present study allows only the identification of associations and does not permit conclusions regarding causal treatment effects or temporal changes in retinal microvascular parameters.

Further prospective longitudinal studies incorporating serial OCTA examinations are needed to establish whether retinal microvascular alterations change over time and whether they reflect treatment response or disease progression [20,24].

*Limitations*: This study has several limitations that should be taken into account. First, the group receiving JAKi included fewer patients than the groups receiving TNFi and IL-17i, which may have affected the statistical power and generalizability of the results. Second, there were significant differences in baseline characteristics between the groups, including age and gender distribution. Patients receiving JAKi were significantly older, and the preponderance of men was more pronounced in the groups receiving IL-17i and JAKi, which may have introduced potential interference factors. In addition, the duration of treatment was significantly shorter in the group of patients receiving JAKi, which may have influenced the observed results and limited the possibility of a direct comparison between the different therapies. Moreover, vessel density measurements in the DCP were not available because of software limitations, which represents an additional limitation of the study. Furthermore, the FAZ area was measured manually, which may have introduced measurement variability. Although all measurements were performed by the same experienced examiner using a standardized protocol, thereby minimizing observer-related variability, intraobserver reproducibility was not formally assessed. Finally, the cross-sectional design of this study limits causal inference and prevents the establishment of temporal relationships between treatment exposure and retinal microvascular changes. Moreover, longitudinal changes in ophthalmic parameters could not be assessed. Therefore, it cannot be determined whether the observed differences are related to treatment exposure, pre-existing intergroup differences, or disease-related factors.

Consequently, the current findings should be regarded as a starting point for formulating hypotheses and require confirmation in prospective longitudinal studies.

In summary, significant differences were observed in retinal microcirculation parameters assessed by OCTA in patients with axSpA treated with various therapeutic agents. The observed differences in retinal microvascular parameters were found among patients receiving different classes of systemic therapy. OCTA appears to be a useful non-invasive imaging modality for assessing retinal microvascular changes in patients with axSpA. Further longitudinal studies involving larger cohorts and automated measurement techniques are needed to confirm these preliminary findings and to clarify the relationship between systemic therapies and retinal microvascular parameters.

## Figures and Tables

**Table 1:** Baseline characteristics of the study participants (*n* = 82 individuals = 159 eyes).

Analyzed Trait	*n* (%)		
No. of participants	82 (n/a)		
No. of eyes	159 (n/a)		
Gender:			
Female	32 (39.02)		
Male	50 (60.98)		
Side:			
Right eye	79 (49.69)		
Left eye	80 (50.31)		
Biological agent:			
TNFi	32 (39.02)	adalimumab, 16 (19.51); certolizumab, 3 (3.66); etanercept, 4 (4.88); golimumab, 9 (10.98)	
IL-17i	30 (36.59)	ixekizumab, 4 (4.88); secukinumab, 26 (31.70)	
JAKi	20 (24.39)	tofacitinib, 1 (1.22); upadacitinib, 19 (23.17)	
History of uveitis	26 (31.71)		
Smoking status	24 (29.27)		
	**M (SD), Me (Q_1_–Q_3_), SE (95% CI)**		
Age (y)	35.72 (7.40)	37 (30–42)	0.58 (34.56–36.88)
T (mmHg)	16.71 (2.46)	17 (15–19)	0.19 (16.33–17.10)
BCVA (logMAR)	1.00 (0.03)	1 (1–1)	0.002 (0.99–1.00)
Superficial FAZ (µm^2^)	255.71 (113.39)	256.09 (188.26–319.57)	8.99 (237.95–273.47)
Deep FAZ (µm^2^)	555.06 (212.64)	528.79 (417.66–672.56)	16.86 (521.75–588.37)
AL (mm)	23.71 (0.97)	23.68 (23.18–24.32)	0.08 (23.56–23.87)
Treatment duration (m)	44.33 (32.05)	34 (23–52)	2.61 (39.17–49.48)

(*n*—number, %—percentage, M—mean, SD—standard deviation, Me—median, Q—quartiles, SE—standard error, CI—confidence interval; TNFi—tumor necrosis factor inhibitor, IL-17i—interleukin 17 inhibitor, JAKi—Janus kinase inhibitor, T—ocular tension, BCVA—best corrected visual acuity, logMAR—logarithm of the minimum angle of resolution, FAZ—foveal avascular zone, AL—axial length.) All qualitative variable variants display percentages calculated with patient-level denominators, except for eye lateralization.

**Table 2:** Baseline characteristics of the study participants’ eyes by therapeutic group (*n* = 82 individuals = 159 eyes).

Analyzed Trait	TNFi	IL-17i	JAKi	*p* Value
	*n* (%) *			
No. of individuals	32 (39.02)	30 (36.59)	20 (24.39)	n/a
No. of eyes	62 (38.99)	58 (36.48)	39 (24.53)	n/a
Gender:				
Female	18 (56.25)	7 (23.33)	7 (35.00)	**0.0269**
Male	14 (43.75)	23 (76.67)	13 (65.00)	
History of uveitis	17 (53.12)	4 (13.33%)	5 (25.00)	**0.0027**
Smoking	10 (31.25)	7 (23.33)	7 (35.00)	0.6419
	**M (SD), Me (Q_1_–Q_3_)**			
Age (y)	34.63 (6.93)	34.91 (7.40)	38.64 (7.54)	**0.0164**
T (mmHg)	16.43 (2.66)	17.01 (2.26)	16.69 (2.41)	0.8315
BCVA (logMAR)	1.00 (0.00)	0.99 (0.04)	1.00 (0.00)	0.2442
Superficial FAZ (µm^2^)	260.68 (96.75)	259.11 (122.74)	242.75 (124.91)	0.8497
Deep FAZ (µm^2^)	600.84 (225.90)	566.52 (180.21)	465.23 (213.23)	**0.0060**
AL (mm)	23.70 (1.06)	23.65 (0.99)	23.83 (0.79)	0.8315
Treatment duration (m)	39 (24–58)	38 (24–65)	27 (19–30)	**0.0007**

* All qualitative variable variants display percentages calculated with patient-level denominators, except the number of eyes examined.

**Table 3:** Descriptive statistics for the density of the superficial plexus vessels [%] in the study participants’ eyes by therapeutic group (*n* = 159 eyes).

Density (%)	TNFi	IL-17i	JAKi	*p* Value
	M (SD)			
Central	18.25 (4.16)	19.34 (4.48)	18.54 (5.26)	0.2846
Superior quadrant	48.56 (1.95)	47.71 (2.87)	48.14 (2.58)	0.1025
Inferior quadrant	48.42 (2.61)	47.89 (3.71)	47.99 (2.09)	0.4370
Nasal quadrant	46.84 (2.17)	45.97 (2.51)	46.64 (2.31)	0.1070
Temporal quadrant	47.74 (2.03)	47.22 (2.33)	47.93 (2.19)	0.1381

M—mean, SD—standard deviation; MT—macular thickness.

**Table 4:** Descriptive statistics for selected measurements in the study participants’ eyes by smoking status (*n* = 159 eyes).

Analyzed Variable	Smokers	Non-Smokers	*p* Value
	M (SD), Me (Q_1_–Q_3_)		
Superficial FAZ (µm^2^)	243.02 (113.09)	261.03 (113.60)	0.2303
Deep FAZ (µm^2^)	493.65 (189.43)	580.83 (217.31)	0.0019
Treatment duration (m)	24 (19–54)	36 (25–52)	0.0386

M—mean, SD—standard deviation; FAZ—foveal avascular zone.

## Data Availability

The raw data supporting the conclusions of this article will be made available by the authors on request.

## References

[1] Katsifis-Nezis D., Fanouriakis A. (2026). Year in Review: Axial Spondyloarthritis. Mediterr. J. Rheumatol..

[2] Ramiro S., Nikiphorou E., Sepriano A., Ortolan A., Webers C., Baraliakos X., Landewé R.B.M., Van den Bosch F.E., Boteva B., Bremander A. (2023). ASAS-EULAR recommendations for the management of axial spondyloarthritis: 2022 update. Ann. Rheum. Dis..

[3] Ghosh N., Ruderman E.M. (2017). Nonradiographic axial spondyloarthritis: Clinical and therapeutic relevance. Arthritis Res. Ther..

[4] Poddubnyy D., Navarro-Compán V., Torgutalp M., Arends S., Aydin S.Z., Battista S., van den Bosch F., Bundy C., Cauli A., Davies J. (2025). The Assessment of SpondyloArthritis International Society (ASAS) consensus-based expert definition of difficult-to-manage, including treatment-refractory, axial spondyloarthritis. Ann. Rheum. Dis..

[5] Walsh J.A., Magrey M. (2021). Clinical Manifestations and Diagnosis of Axial Spondyloarthritis. J. Clin. Rheumatol..

[6] Beringer A., Miossec P. (2019). Systemic effects of IL-17 in inflammatory arthritis. Nat. Rev. Rheumatol..

[7] Ursini F., Leporini C., Bene F., D’Angelo S., Mauro D., Russo E., de Sarro G., Olivieri I., Pitzalis C., Lewis M. (2017). Anti-TNF-alpha agents and endothelial function in rheumatoid arthritis: A systematic review and meta-analysis. Sci. Rep..

[8] Łosińska K., Korkosz M., Kwaśny-Krochin B. (2019). Endothelial dysfunction in patients with ankylosing spondylitis. Reumatologia.

[9] Karbach S., Croxford A.L., Oelze M., Schüler R., Minwegen D., Wegner J., Koukes L., Yogev N., Nikolaev A., Reißig S. (2014). Interleukin 17 drives vascular inflammation, endothelial dysfunction, and arterial hypertension in psoriasis-like skin disease. Arterioscler. Thromb. Vasc. Biol..

[10] Raychaudhuri S.P., Shah R.J., Banerjee S., Raychaudhuri S.K. (2024). JAK-STAT Signaling and Beyond in the Pathogenesis of Spondyloarthritis and Their Clinical Significance. Curr. Rheumatol. Rep..

[11] Webers C., Ortolan A., Sepriano A., Falzon L., Baraliakos X., Landewé R.B.M., Ramiro S., van der Heijde D., Nikiphorou E. (2023). Efficacy and safety of biological DMARDs: A systematic literature review informing the 2022 update of the ASAS-EULAR recommendations for the management of axial spondyloarthritis. Ann. Rheum. Dis..

[12] Klavdianou K., Papagoras C., Baraliakos X. (2023). JAK Inhibitors for the Treatment of Axial Spondyloarthritis. Mediterr. J. Rheumatol..

[13] Benavent D., Navarro-Compán V. (2024). Exploring the latest advances in axial spondyloarthritis management. Nat. Rev. Rheumatol..

[14] Bautista-Molano W., Fernández-Ávila D.G., Brance M.L., Ávila Pedretti M.G., Burgos-Vargas R., Corbacho I., Cosentino V.L., Díaz Coto J.F., Giraldo Ho E., Gomes Resende G. (2023). Pan American League of Associations for Rheumatology recommendations for the management of axial spondyloarthritis. Nat. Rev. Rheumatol..

[15] Deng R., Jiang X., Zhong X.-X., Shen F., Qian F., Quan C.-W. (2026). Current status and emerging trends in biological therapies for ankylosing spondylitis: A bibliometric analysis (2004–2024). Medicine.

[16] Li D., Zhang X., Tian Y., Zhang J., Zhao X., Li M., Zhao Y., Liu X. (2025). Efficacy and Safety of Tumor Necrosis Factor Inhibitors, Interleukin-17 Inhibitors, and Janus Kinase Inhibitors in Patients with Non-Radiographic Axial Spondyloarthritis: A Systematic Review and Network Meta-Analysis. Int. Arch. Allergy Immunol..

[17] Jia Y., Tan O., Tokayer J., Potsaid B., Wang Y., Liu J.J., Kraus M.F., Subhash H., Fujimoto J.G., Hornegger J. (2012). Split-spectrum amplitude-decorrelation angiography with optical coherence tomography. Opt. Express.

[18] Paczwa K., Golebiewska J. (2020). Optical Coherence Tomography and Optical Coherence Tomography Angiography in Ophthalmology. Pol. J. Aviat. Med. Bioeng. Psychol..

[19] Waizel M., Todorova M.G., Terrada C., LeHoang P., Massamba N., Bodaghi B. (2018). Superficial and deep retinal foveal avascular zone OCTA findings of non-infectious anterior and posterioruveitis. Graefes Arch. Clin. Exp. Ophthalmol..

[20] Samanci R., Meydan B., Teberik K., Meydan S.U., Ataoglu S. (2026). Retinal microvascular changes in patients with ankylosing spondylitis receiving biologic therapy: A cross-sectional OCTA study. BMC Ophthalmol..

[21] Danve A., Deodhar A. (2022). Treatment of axial spondyloarthritis: An update. Nat. Rev. Rheumatol..

[22] Zhu X., Yang K., Xiao Y., Ye C., Zheng J., Su B., Zheng Y., Zhang X., Shi K., Li C. (2022). Association of Cigarette Smoking with Retinal Microvascular Changes Assessed by Optical Coherence Tomography Angiography in a Large Population-Based Study. Acta Ophthalmol..

[23] Gokmen O., Ozgur G. (2023). Effects of Long-Term Cigarette Smoking on Retinal and Choroidal Microvasculature Assessed by Optical Coherence Tomography Angiography. Eur. J. Ophthalmol..

[24] Çiloğlu E., Unal F., Sukgen E.A., Kocluk Y., Dogan N.C. (2020). Evaluation of the Effect of Chronic Smoking on Retinal Microvascular Density and Foveal Avascular Zone Using Optical Coherence Tomography Angiography. J. Curr. Ophthalmol..

